# Cell Wall Integrity Mediated by CfCHS1 Is Important for Growth, Stress Responses and Pathogenicity in *Colletotrichum fructicola*

**DOI:** 10.3390/jof9060643

**Published:** 2023-06-01

**Authors:** Rongcun Gan, Shengpei Zhang, He Li

**Affiliations:** 1 Key Laboratory of National Forestry, Grassland Administration on Control of Artificial Forest Diseases and Pests in South China, Central South University of Forestry and Technology, Changsha 410004, ChinaT20182400@csuft.edu.cn (S.Z.); 2 Hunan Provincial Key Laboratory for Control of Forest Diseases and Pests, Central South University of Forestry and Technology, Changsha 410004, China

**Keywords:** chitin synthase, conidiation, pathogenicity, *C. fructicola*

## Abstract

*Camellia oleifera*, a woody plant that produces edible oil, is indigenous to China. The devastating disease of anthracnose inflicts significant financial losses on *Ca. oleifera*. The primary causative agent of anthracnose on *Ca. oleifera* is *Colletotrichum fructicola*. Chitin, a pivotal constituent of fungal cell walls, assumes a critical function in their proliferation and maturation. To study the biological functions of chitin synthase 1(Chs1) in *C. fructicola*, the *CfCHS1* gene knockout mutants, ∆*Cfchs1*-1 and ∆*Cfchs1*-2, and their complementary strain, ∆*Cfchs1/CfCHS1*, of *C. fructicola* were generated. Our results showed that the colony diameters of wild-type and complement-strain ∆*Cfchs1/CfCHS1*, mutant ∆*Cfchs1*-1 and ∆*Cfchs1*-2 cultured on the CM and MM medium were 5.2, 5.0, 2.2 and 2.4 cm and 4.0, 4.0, 2.1 and 2.6 cm, respectively, which were significantly smaller for the mutant than for the wild type and complement strain; the inhibition rates on the CM medium supplemented with H_2_O_2_, DTT, SDS and CR were 87.0% and 88.5%, 29.6% and 27.1%, 88.0% and 89.4%, and 41.7% and 28.7%, respectively, for the mutant strains, ∆*Cfchs1*-1 and ∆*Cfchs1*-2, which were significantly higher than those for the other two strains; the rate of hyphal tips with CFW fluorescence in ∆*Cfchs1*-1 and ∆*Cfchs1*-2 was 13.3% and 15.0%, which was significantly lower than those for the other two strains; the mutant strains, ∆*Cfchs1*-1 and ∆*Cfchs1*-2, lost the ability to produce conidia; the mutant strains showed weaker pathogenicity on wounded and unwounded *Ca. oleifera* leaves than the wild type and complement strain. The findings of this study suggest that CfChs1 plays a crucial role in the growth and development, stress responses, and pathogenicity of *C. fructicola*. Thus, this gene could be a potential target for developing novel fungicide.

## 1. Introduction

In southern China, *Camellia oleifera* has been cultivated for more than 2000 years as an edible-oil plant [1]. The *Ca*. *oleifera* industry has developed rapidly in recent years. It is known that anthracnose generally occurs in the oil-tea trees of China, which cause a great deal of damage each year to the trees [2]. Anthracnose on *Ca*. *oleifera* is commonly thought to be caused by the *Colletotrichum gloeosporioides* complex [3]. It has been found that the pathogens of anthracnose include *Colletotrichum fructicola*, *C. siamense*, *C. gloeosporioides*, *C. camelliae*, *C. horii*, *C. karstii*, *C. henanense*, *C. nymphaeae* and *C. aeschynomenes* [2,4,5]. Among these pathogens, *C. fructicola* is the dominant species causing anthracnose on *Ca*. *oleifera* [2].

In pathogenic fungi, the cell wall plays an important role during host invasion [6]. Chitin, a β (1,4)-linked homopolymer of N-acetylglucosamine, is an important component of fungal cell walls [7]. Chitin is synthesized by a large family of chitin synthase (CHS) [7]. Different chitin synthases produce chitin localized to specific cell-wall-derived structures or developmental stages [8,9,10]. It has been reported that the number and function of chitin synthase are different in fungi [7,11].

In *Saccharomyces cerevisiae*, three classes of chitin synthases were identified, with distinct functions in cell wall expansion, septum formation and budding [12,13,14,15,16]. Chs1 (class I) is involved in chitin repair at the end of cytokinesis; Chs2 (class II) is responsible for the synthesis of the primary septum and cell division; Chs3 (class IV) is required for the formation of chitin rings at the base of emerging buds and chitin synthesis in lateral cells [16]. Although class III of chitin synthase is not identified in yeast, it is involved in pathogenicity in a large number of plant pathogenic fungi [9,17,18,19,20,21,22]. In *Magnaporthe oryzae*, MoChs1 (class III) plays an important role in conidiation, response to oxidative stress, appressorium formation and plant infection [17].

The function of chitin synthase in forest fungal pathogens including *C. fructicola* is unknown. In the *C. fructicola* genome, seven *CHS* gene clusters were predicted. This article focuses on class III chitin synthase Chs1 in *C. fructicola*, because CfChs1 plays an important role in virulence. Here, we characterized the function of Chs1 in *C. fructicola* for the first time.

## 2. Materials and Methods

### 2.1. Strains and Culture Conditions

*C. fructicola* CFLH16 was used as the wild-type strain. All strains were cultured on CM plates at 28 °C in the darkness, unless indicated otherwise. The strains were cultured in liquid CM plates in darkness, shaken at 28 °C for 2 days and collected for the extraction of genomic DNA.

### 2.2. Phylogenetic Tree Construction and Domain Prediction

The class III chitin synthase in *C. liriopes*, *C. siamense*, *C. fructicola*, *F. graminearum*, *M. oryzae*, *B. cinerea*, *N. crassa* and *A. nidulans* were obtained from the NCBI database (https://www.ncbi.nlm.nih.gov/, accessed on 12 March 2023). The MEGA 7.0 program with the neighbor-joining method was used to construct the phylogenetic tree. The domains of CfChs1 were predicted using the SMART websites (http://smart.embl-heidelberg.de/, accessed on 12 March 2023).

### 2.3. Gene Deletion and Complementation Assays

The targeted gene deletion was accomplished using the homologous recombination method described by Zhang et al. [3]. Complementation assays were performed as described by Zhang et al. [23].

### 2.4. Growth and Conidiation Assays

The strains were cultured on CM and MM agar plates at 28 °C in the darkness for 3 days, and the colony diameters were measured and statistically analyzed. For conidiation assays, the strains were cultured in liquid CM with plate shaking for 3 days; then, they were filtered with three layers of lens paper and observed and quantified on a microscope.

### 2.5. Stress Response Assays

The strains were cultured on CM and CM plus with various stresses, including cell wall stress (400 μg·ml^−1^ CR and 0.01% SDS), oxidative stress (5.0 mM H_2_O_2_) and ER stress (5.0 mM DTT). After 3 days of incubation, the colony diameters were measured and the inhibition rates were statistically analyzed.

### 2.6. Pathogenicity Assays

The mycelial plugs of the strains were inoculated onto the edge of unwounded and wounded *Ca. oleifera* leaves. The inoculated leaves were kept in a humid condition at room temperature. After incubation for 3–5 days, the lesions were observed and photographed.

### 2.7. CFW Staining

The mycelia were incubated in liquid CM for 1 day, followed by filtration. The hyphae of the strains were further stained with CFW (10 µg·mL^−1^) in the dark for 5 min. After being washed twice with ddH_2_O, the blue fluorescent signals were observed under a microscope.

### 2.8. Statistical Analysis

All results were expressed as the mean ± SD of three replicates. The statistical data were analyzed via ANOVA (Analysis of Variance) with Duncan’s new multiple range test.

## 3. Results

### 3.1. Identification and Phylogenetic Analysis of CfChs1 in C. fructicola

**Figure 1:**
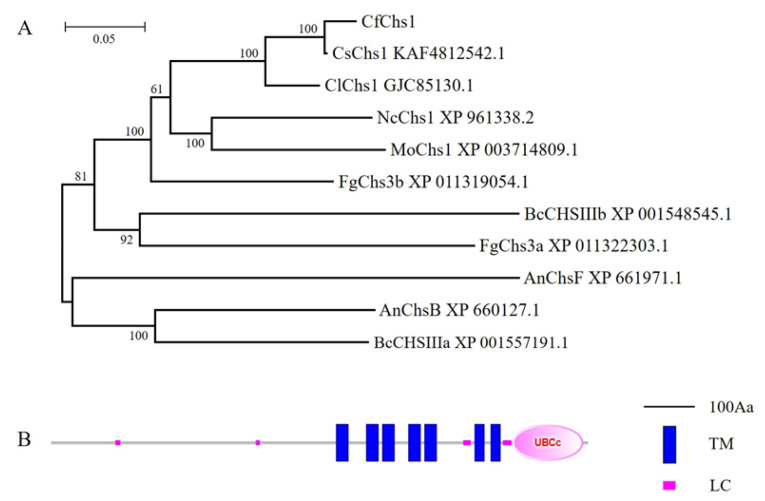
Phylogenetic analysis and domain prediction of CfChs1. (**A**): The class III chitin synthases from diverse fungi were aligned using the CLUSTAL_W, and the neighbor-joining tree was constructed using MEGA 7.0 with 1000 bootstrap replicates. Corresponding species name: Cf: *Colletotrichum fructicola*; Cl: *Colletotrichum liriopes*; Cs: *Colletotrichum siamense*; Fg: *Fusarium graminearum*; Mo: *Magnaporthe oryzae*; Nc: *Neurospora crassa*; Bc: *Botrytis cinerea*; An: *Aspergillus nidulans*. (**B**): The domains of CfChs1 were predicted. TM: transmembrane domain; LC: low-complexity region; UBCc: ubiquitin-conjugating enzyme E2 domain.

Using the *M. oryzae* Chs1 amino acid sequence as the trace, we identified Chs1 in the *C. fructicola* genome database and named it CfChs1. According to the classification method of Chigira [24] and Choquer [25], CfChs1 is considered to be a class III chitin synthase. CfChs1 was predicted to encode 1070 amino acids. The phylogenetic dendrogram revealed that CfChs1 shows sequence conservation among other fungi class III chitin synthases; CfChs1 shows higher amino acid sequence homology with that of *C. siamense* (94.87% identities) (Figure 1A). The domain prediction using the SMART website (http://smart.embl-heidelberg.de/, accessed on 12 March 2023) suggested that CfChs1 contains seven transmembrane (TM) domains, a ubiquitin-conjugating enzyme E2 (UBCc) domain and four low-complexity regions (Figure 1B).

### 3.2. Targeted Deletion of CfCHS1 Gene in C. fructicola

To characterize the functions of CfChs1, the coding region of *CfCHS1* was replaced with the *HPH* gene according to the homologous recombination principle (Figure S1A). Putative transformants were screened on hygromycin media and verified via PCR amplification. We thus acquired the *CfCHS1* gene deletion mutant ∆*Cfchs1*-1 and ∆*Cfchs1*-2 (Figure S1B). These two mutants showed the same biological phenotypes. Moreover, the ∆*Cfchs1*-1 mutant was also complemented with the wild-type *CfCHS1* gene that restored all defects.

### 3.3. The CfChs1 Regulates Vegetative Growth

**Figure 2:**
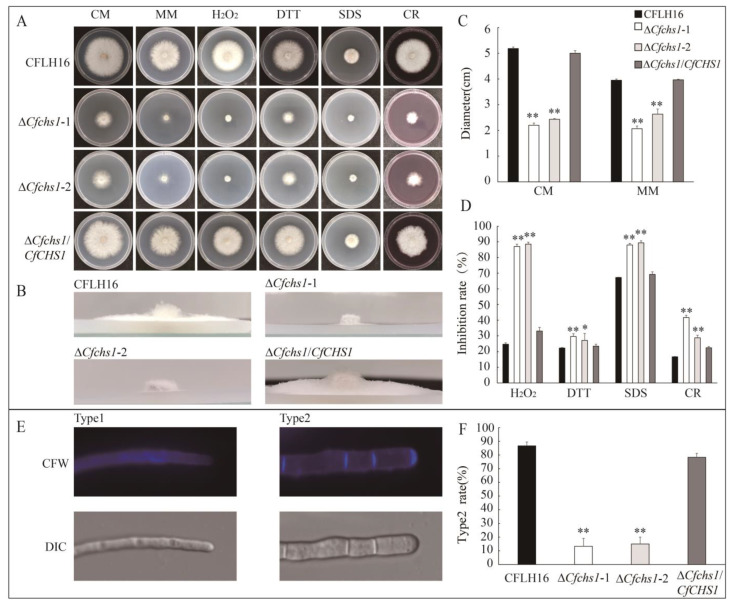
CfChs1 is involved in vegetative growth, environmental stress tolerance and cell wall integrity. (**A**): Growth rate of strains on CM, MM and CM medium with various stress agents added. (**B**): Aerial hyphae growth is observed on MM medium. (**C**): Statistical analysis of the colony diameter variations. (**D**): Growth inhibition rate of strains in different environmental stress media. (**E**): The mycelia of the strains were stained with 10 µg/mL CFW for 5 min without light before being photographed. The experiment was repeated three times with triplicates, which showed the same results. DIC, differential interference contrast image. Type1 represents chitin not accumulated in mycelial tips; Type2 represents chitin accumulated in mycelial tips. (**F**): The ratio of chitin accumulation at hyphal tip. Error lines are standard deviations, **: indicates highly significant difference (*p* < 0.01), and *: indicates significant difference (*p* < 0.05).

To investigate the effect of CfChs1 in vegetative development, wild-type (WT), ∆*Cfchs1*-1, ∆*Cfchs1*-2 and complemented-strain ∆*Cfchs1/CfCHS1* were cultured in plates of CM and MM media for 3 days. The results demonstrated that the colony diameters of WT, complement-strain ∆*Cfchs1/CfCHS1*, mutant ∆*Cfchs1*-1 and ∆*Cfchs1*-1 cultured on the CM and MM media were 5.2, 5.0, 2.2, 2.4 cm and 4.0, 4.0, 2.1, 2.6 cm. Compared with the WT, ∆*Cfchs1*-1 and ∆*Cfchs1*-2 showed significant reduced growth rates, while ∆*Cfchs1/CfCHS1* compensated for their defect (Figure 2A,C). Moreover, we also found that ∆*Cfchs1*-1 and ∆*Cfchs1*-2 showed reduced aerial hyphal growth in the MM plate, which exhibited a flat colony compared with the fluffy colony of WT and ∆*Cfchs1/CfCHS1* (Figure 2B). However, ∆*Cfchs1*-1 and ∆*Cfchs1*-2 did not show significant reduced aerial hyphal growth in the CM medium.

### 3.4. The Role of CfChs1 in Oxidative and ER Stress and Cell Wall Integrity

For normal growth and infection, fungi must undergo many types of stress in nature, such as oxidative stress, endoplasmic reticulum stress, cell wall stress, etc. To address the role of CfChs1 in environmental adaptation, here, we investigated the roles of CfChs1 in the response to environmental stresses. We cultured the WT, ∆*Cfchs1*-1, ∆*Cfchs1*-2 and complemented-strain ∆*Cfchs1/CfCHS1* on CM plates and CM plates supplemented with stress for 3 days.

We found that the average inhibition rates on the CM medium supplemented with oxidative stress (5 mM H_2_O_2_), endoplasmic reticulum stress (5 mM DTT) and cell wall stress (0.01% SDS and 400 μg·ml^−1^ CR) were 87.0% and 88.5%, 29.6% and 27.1%, 88.0% and 89.4%, and 41.7% and 28.7% for the mutant ∆*Cfchs1*-1 and ∆*Cfchs1*-1, which were significantly higher than those for the WT. In addition, ∆*Cfchs1/CfCHS1* compensated for these defects (Figure 2A,D). This result indicated that CfChs1 participates in the response to oxidative, ER and cell wall stresses in *C. fructicola*.

Cell wall integrity inhibitors (CFW and CR) bind to nascent chitin chains and inhibit the assembly enzymes that connect chitin to β-1,3-glucan and β-1,6-glucan [26]. We characterized the importance of CfChs1 in cell wall stress tolerance. In order to further study the role of CfChs1 in maintaining cell wall integrity in *C. fructicola*, we used CFW as a chitinous fluorochrome to stain the hyphae to assess the distribution of chitinous substances at the tip of hyphae. We noted that there were two types of mycelia in WT, ∆*Cfchs1*-1, ∆*Cfchs1*-2 and ∆*Cfchs1/CfCHS1*, in one of which, CFW fluorescence was mostly accumulated at the growing apices and another one did not show obvious fluorescence at the hyphal tip (Figure 2E). The rate of hyphal tips with fluorescence in ∆*Cfchs1*-1 and ∆*Cfchs1*-2 was 13.3% and 15.0%, while in WT or ∆*Cfchs1/CfCHS1*, it was above 78%. These results indicated that CfChs1 plays crucial roles in maintaining cell wall integrity (Figure 2F). We speculated that the deficiency of chitin synthase in cell wall integrity was the main reason for the stress sensitivity changing in ∆*Cfchs1*.

### 3.5. The CfChs1 Is Essential for Pathogenicity and Conidiation

**Figure 3:**
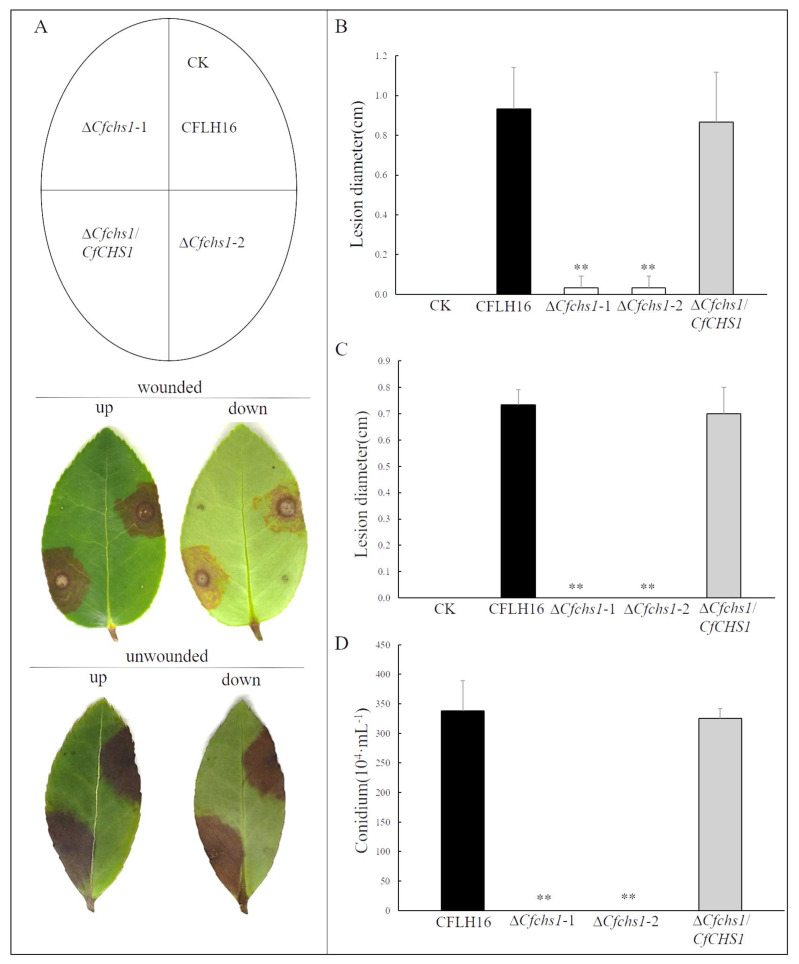
CfChs1 is essential for pathogenicity and conidiation. (**A**): Unwounded and wounded *Ca. oleifera* leaves were inoculated with mycelial plugs of strains. Up, up of the leaf; Down, down of the leaf. CK: compared with the control, the agar plug was inoculated onto it. (**B**): Statistical analysis of the lesion diameter on wounded leaves. (**C**): The lesion diameter on unwounded leaves. (**D**): Conidium formation rate. Error lines are used as standard deviations; **: indicates highly significant differences (*p* < 0.01).

To characterize the roles of CfChs1 in pathogenicity, mycelial plugs of WT, ∆*Cfchs1*-1, ∆*Cfchs1*-2 and complemented-strain ∆*Cfchs1/CfCHS1* were inoculated on wounded *Ca. oleifera* leaves. After 3 days, the lesion diameter in the mutant-strain ∆*Cfchs1*-1 and ∆*Cfchs1*-2 was significantly lower than in the WT and ∆*Cfchs1/CfCHS1* (Figure 3A,B). Moreover, we also conducted a pathogenicity assay on unwounded *Ca. oleifera* leaves. We found that ∆*Cfchs1* produced no lesions, in contrast to the typical lesions caused by WT and ∆*Cfchs1/CfCHS1* (Figure 3A,C). Based on the above observations, CfChs1 plays a crucial role in pathogenesis.

In plant-pathogenic fungi, conidia play an important role in the disease cycle [27,28]. To examine the role of CfChs1 in conidiation, WT, ∆*Cfchs1*-1, ∆*Cfchs1*-2 and complemented-strain ∆*Cfchs1/CfCHS1* were cultured in liquid CM with shaking for 3 days. We found that ∆*Cfchs1*-1 and ∆*Cfchs1*-2 did not produce conidia, while the WT and ∆*Cfchs1/CfCHS1* produced a large number of conidia (Figure 3D). This result demonstrated that CfChs1 was important for conidiation in *C. fructicola*.

## 4. Discussion

The main enzyme involved in chitin synthesis is chitin synthase (CHS) [6,11]. The class III chitin synthase gene, *CHS1*, is important for maintaining cell wall integrity. In the present study, we characterized CfChs1, a class III chitin synthase, as the homolog of *M. oryzae* Chs1 in *C. fructicola*. We found that CfChs1 plays critical roles in vegetative growth, conidiation, stress responses and pathogenicity in *C. fructicola*.

Mycelial biomass is a significant parameter to directly evaluate growth. The deletion of the *CfCHS1* gene in *C. fructicola* resulted in a significant decrease in the mycelial growth rate. The aerial hyphae of the mutant ∆*Cfchs1* on CM medium did not change significantly, while almost no aerial hyphae were produced on MM medium, indicating that nutritional conditions determine the ability of ∆*Cfchs1* to produce aerial hyphae. CfChs1 is involved in regulating the vegetative growth in *C. fructicola*. This is similar to the function of class III chitin synthase AnChsB in *A. nidulans* and NcChs1 in *N. crassa* [29,30]. However, the two class III chitin synthases, FgChs3a and FgChs3b, in *F. graminearum* showed great differences. The FgChs3b knockout mutant was unable to grow, while the deletion of FgChs3a had no effects on vegetative growth [9]. In addition, the class III chitin synthase MoChs1 also had no significant effect on the vegetative growth of *M. oryzae* [17]. These results showed that the effect of Chs1 on fungal growth varies among species.

As conidia is very important for the spread of phytopathogenic fungus, we next assessed the role of CfChs1 in conidiation. ∆*Cfchs1* was cultured in CM liquid medium for 3 days, and no conidia were observed. This result is consistent with previous reports that AnchsB (homologous to CHS1) was involved in conidiation in *A. nidulans* [31]. In *M. oryzae*, the effect of MoChs1 on conidiation is not only manifested in a decrease in quantity but also in morphological abnormalities [17]. The specific mechanism of the CfChs1 regulation of conidiation requires further investigation.

An important characteristic of plant-pathogenic fungus is its pathogenicity. The previous study found that MoChs1 (class III) and BcChs3a (class III) are involved in pathogenicity [17,21]. In *C. fructicola*, the deletion of the *CfCHS1* gene resulted in a significant decrease in the pathogenicity on wound or unwounded leaves of *Ca. oleifera*. These results comply with other studies suggesting the significant contribution of the *CHS1* gene in the pathogenicity of phytopathogenic fungus.

The cell wall is critical for the virulence of fungi. The sensitivity of ∆*Cfchs1* to environmental stress was then assessed. The mutant decreased the ability to survive diverse environmental stresses, including exposure to oxidative stress, cell wall stress and endoplasmic reticulum stress. In this study, a prominent apical chitin cap was rarely found in ∆*Cfchs1* mutant hyphae, suggesting that CfChs1 participates in chitin synthesis at the hyphal apex. This may be the main reason for the pathogenicity defect of ∆*Cfchs1*. A reasonable hypothesis to account for this phenotype is that the CfChs1 of *C. fructicola* is an essential protagonist of chitin synthesis at the hyphal apex and is involved in polarized growth at hyphal tips.

Our results demonstrated that CfChs1 is essential for maintaining cell wall integrity.

CfChs1 deficiency affected the *C. fructicola* cell wall composition, which is the main reason for the loss of pathogenicity of ∆Cfchs1 mutants. However, the specific mechanism is unclear and warrants further studies.

## 5. Conclusions

*Colletotrichum fructicola* is the major pathogen of anthracnose on *Ca. oleifera*. In this study, the *CfCHS1* gene was identified in the *C. fructicola* genome via a homology-search method. Our research showed that CfChs1 was involved in vegetative growth, asexual development, stress responses and the pathogenicity of *C. fructicola*. Thus, the *CfCHS1* gene could be a potential target for developing novel fungicide.

## Data Availability

All data supporting the findings of this study are available within the paper and within its supplementary materials published online.

## Supplementary Materials

The following supporting information can be downloaded at: https://www.mdpi.com/article/10.3390/jof9060643/s1, Figure S1. Generation of the *CfCHS1* gene deletion mutant in *C. fructicola*. (**A**) Schematic illustration for deletion strategy of *CfCHS1* gene. (**B**) Validation of the *CfCHS1* gene deletion mutant. Table S1. The primers used in this study.

## Abbreviations

PCR (polymerase chain reaction); CR (Congo red); SDS (sodium dodecyl sulfate); DTT (DL-Dithiothreitol); CFW (Calcofluor white); complete medium (CM); minimal medium (MM).
