# Supplementary material for: Cell Wall Integrity Mediated by CfCHS1 Is Important for Growth, Stress Responses and Pathogenicity in Colletotrichum fructicola
Source: J Fungi (Basel). 2023 Jun 1;9(6):643. doi: 10.3390/jof9060643 (PMC10305008; doi:10.3390/jof9060643)
Supplement: Supplementary file 1 [file jof-09-00643-s001.zip › jof-2337866-supplementary.pdf]

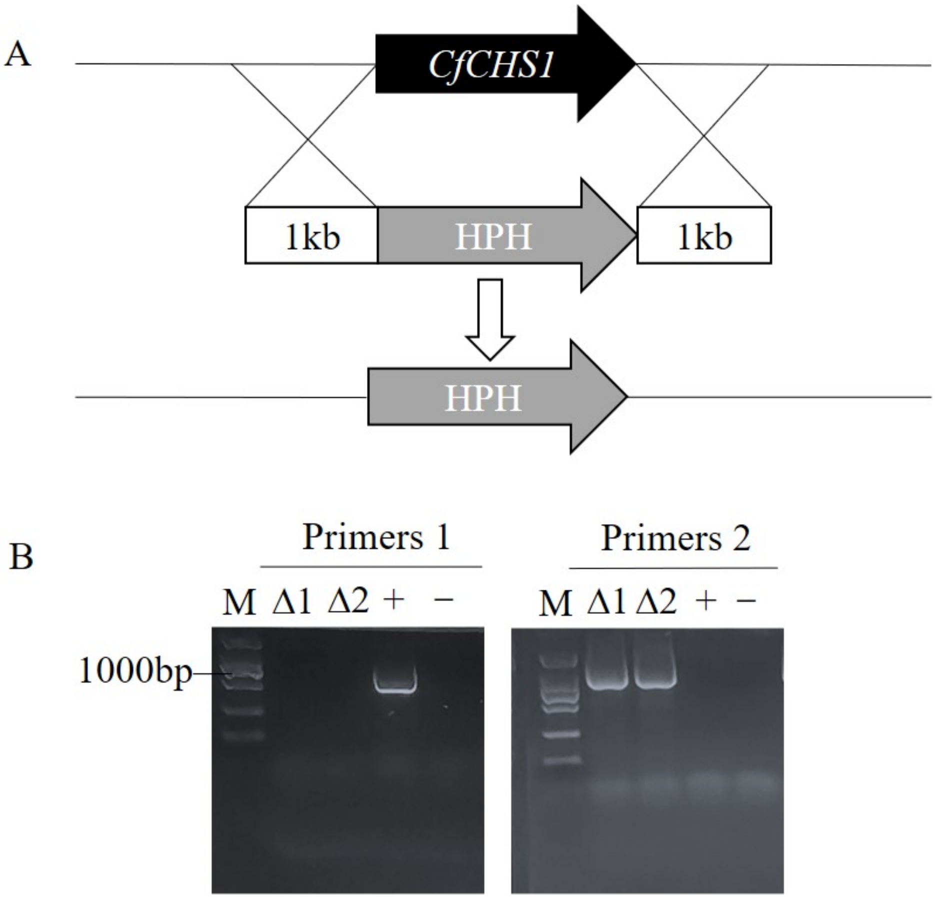

Figure S1. Generation of the *CfCHS1* gene deletion mutant in *C. fructicola*. (A) Schematic illustration for deletion strategy of *CfCHS1* gene. (B) Validation of the *CfCHS1* gene deletion mutant.

Table S1. Primers used in this study

Primer	Sequence (5′-3′)
CHS1-1F	TGCTCTCTGGTGTGCATCTT
CHS1-2R	TTGACCTCCACTAGCTCCAGCCAAGCCGTTTGCTGCGGGTGAACTCA
CHS1-3F	CAAAGGAATAGAGTAGATGCCGACCGGATGGCCATCTGCAAATAGA
CHS1-4R	CTGTGCTCTTGTGTGTCCAT
CHS1-5F	TCTGCTGATGTCTGATGTCG
CHS1-7F	CTGTGGCAAGCCATATTCGC
CHS1-8R	GGCCGACAATAACGGCATTC
CHS1-9F	ACTCACTATAGGGCGAATTGGGTACTCAAATTGGTTCAAGTATGGTGTCCAGGAGA
CHS1-10R	CACCACCCCGGTGAACAGCTCCTCGCCCTTGCTCAC TTTCAACACACGGTCGTAGC
Hyg F	GGCTTGGCTGGAGCTAGTGGAGGTCAA
Hyg R	CGGTCGGCATCTACTCTATTCCTTTG
GFP-R	GACACGCTGAACTTGTGGCCGTT
H855R	GCTGATCTGACCAGTTGC
